# Comparative genomic and transmission analysis of *Clostridioides difficile* between environmental, animal, and clinical sources in China

**DOI:** 10.1080/22221751.2021.2005453

**Published:** 2021-12-01

**Authors:** Yanzi Zhou, Wangxiao Zhou, Tingting Xiao, Yunbo Chen, Tao Lv, Yuan Wang, Shuntian Zhang, Hongliu Cai, Xiaohui Chi, Xiaoyang Kong, Kai Zhou, Ping Shen, Tongling Shan, Yonghong Xiao

**Affiliations:** a State Key Laboratory for Diagnosis and Treatment of Infectious Diseases, National Clinical Research Center for Infectious Diseases, Collaborative Innovation Center for Diagnosis and Treatment of Infectious Diseases, the First Affiliated Hospital, Zhejiang University School of Medicine, Hangzhou, People’s Republic of China; b Department of Intensive Care Unit, the First Affiliated Hospital, Zhejiang University School of Medicine, Hangzhou, People’s Republic of China; c Shenzhen Institute of Respiratory Diseases, the First Affiliated Hospital (Shenzhen People’s Hospital), Southern University of Science and Technology, and Second Clinical Medical College, Jinan University, Shenzhen, People’s Republic of China; d Department of Swine Infectious Diseases, Shanghai Veterinary Research Institute, Chinese Academy of Agricultural Sciences, Shanghai, PR People’s Republic of China.

**Keywords:** *Clostridioides difficile* infection, asymptomatic carrier, multiple sources, comparative genome, transmission

## Abstract

*Clostridioides difficile* is the most common pathogen causing antibiotic-associated diarrhea. Previous studies showed that diverse sources, aside from *C. difficile* infection (CDI) patients, played a major role in *C. difficile* hospital transmission. This study aimed to investigate relationships and transmission potential of *C. difficile* strains from different sources. A prospective study was conducted both in the intensive care unit (ICU) and six livestock farms in China in 2018–2019. Ninety-eight strains from CDI patients (10 isolates), asymptomatic hospitalized carriers (55), the ICU environment (12), animals (14), soil (4), and farmers (3) were collected. Sequence type (ST) 3/ribotype (RT) 001, ST35/RT046, and ST48/RT596 were dominant types, distributed widely in multiple sources. Core-genome single-nucleotide polymorphism (cgSNP) analysis showed that hospital and farm strains shared several common clonal groups (CGs, strains separated by ≤ 2 cgSNPs) (CG4/ST3/RT001, CG7/ST35/RT046, CG11/ST48/RT596). CDI patients, asymptomatic carriers, and the ICU environment strains also shared several common CGs. The number of virulence genes was not statistically different between strains from different sources. Multi-source strains in the same CG carried identical virulence gene sequences, including pathogenicity genes at the pathogenicity locus and adhesion-related genes at S-layer cassette. Resistance genes (*ermB*, *tetM*, etc.) were widespread in multiple sources, and multi-source strains in the same CG had similar resistance phenotypes and carried consistent transposons and plasmid types. The study indicated that interspecies and cross-regional transmission of *C. difficile* occurs between animals, the environment, and humans. Community-associated strains from both farms and asymptomatic hospitalized carriers were important reservoirs of CDI in hospitals.

## Introduction

*Clostridioides difficile*, a spore-forming, Gram positive, anaerobic bacterium, is the most common pathogen causing antibiotic-associated diarrhea [1], and responsible for 15–25% of infectious diarrhea cases, which can lead to serious complications, including life-threatening pseudomembranous colitis and toxic megacolon [2]. Antimicrobial therapy, advanced age (>65 years), and longer hospital stays are the major risk factors for healthcare-associated *C. difficile* infection (HA-CDI) [3]. In recent years, the incidence of community-associated CDI (CA-CDI) increased globally, with CA-CDI accounting for approximately 41% of all CDI cases in the United States and nearly 30% of CDI cases in Australia [4, 5], which suggests that CDI is no longer just a healthcare-associated event.

For a long time, studies on *C. difficile* as an important pathogen causing healthcare-associated infections were limited to patients with CDI, and researchers attempted to control *C. difficile* dissemination in healthcare institutions by focusing on patients with CDI. However, several studies showed that enhanced control measures, such as contact precaution and isolation, still have limited efficacy in reducing the prevalence of CDI in hospitals [6]. Making use of whole-genome sequencing (WGS), some investigations found that most CDI cases were genetically distinct, suggesting that diverse sources, in addition to patients with CDI, such as asymptomatic carriers, environment, play a major role in *C. difficile* transmission in hospitals [7]. Riggs et al. [6] found that asymptomatic hospitalized carriers are a potential source for transmission of epidemic and non-epidemic CDI strains among long-term care facility residents. The prevalence of asymptomatic carriers in hospitals was much greater than that of CDI [8], and *C. difficile* strains isolated from newly admitted patients were community associated [9]. Notably, asymptomatic carriers carrying toxigenic *C. difficile* are six times more likely to develop CDI after admission to the hospital than non-carriers, thus becoming a direct source of CDI [10].

With increasing research focus on CA-CDI, researchers found that *C. difficile* can not only be recovered from gastrointestinal tracts of humans and animals, but even from natural, healthcare, and domestic environments, as well as from food products, such as meat and vegetables [11, 12]. As the spore form being metabolically inactive and resistant to heat, desiccation, and the alcohol-based solutions used for hand hygiene in hospital [13], *C. difficil*e can persist in diverse sources for a long time, which might be potential reservoirs of CDI strains. The most common *C. difficile* strains isolated from animals are sequence type (ST) 11/ribotype (RT) 078 in Europe and the United States, and RT014 in Australia, and both lineages can transmit between humans and animals [14]. Another study carried out in Sweden found that ST35/RT046 was transmitted between pigs and humans [15]. There are limited data on the transmission of *C. difficile* from diverse sources in other ST/RT lineages.

However, studies on *C. difficile* in non-human hosts are rare in China, all of which are limited to epidemiological investigations [16, 17]. There is a lack of studies on genomic characteristics and transmission patterns of *C. difficile* strains from multiple sources such as animals and the environment. In this study, we collected stool samples from CDI patients, asymptomatic carriers, farmers, animals, and swab samples from the intensive care unit (ICU) environment and the farm environment. *C. difficile* strains were isolated and subjected to WGS. Molecular epidemiology and comparative genomic analyses were performed to investigate the relationship between and transmission potential of *C. difficile* strains from different sources, thus exploring the transmission pattern.

## Materials and methods

### Patient enrollment and environmental and animal sampling.

**Figure 1.**
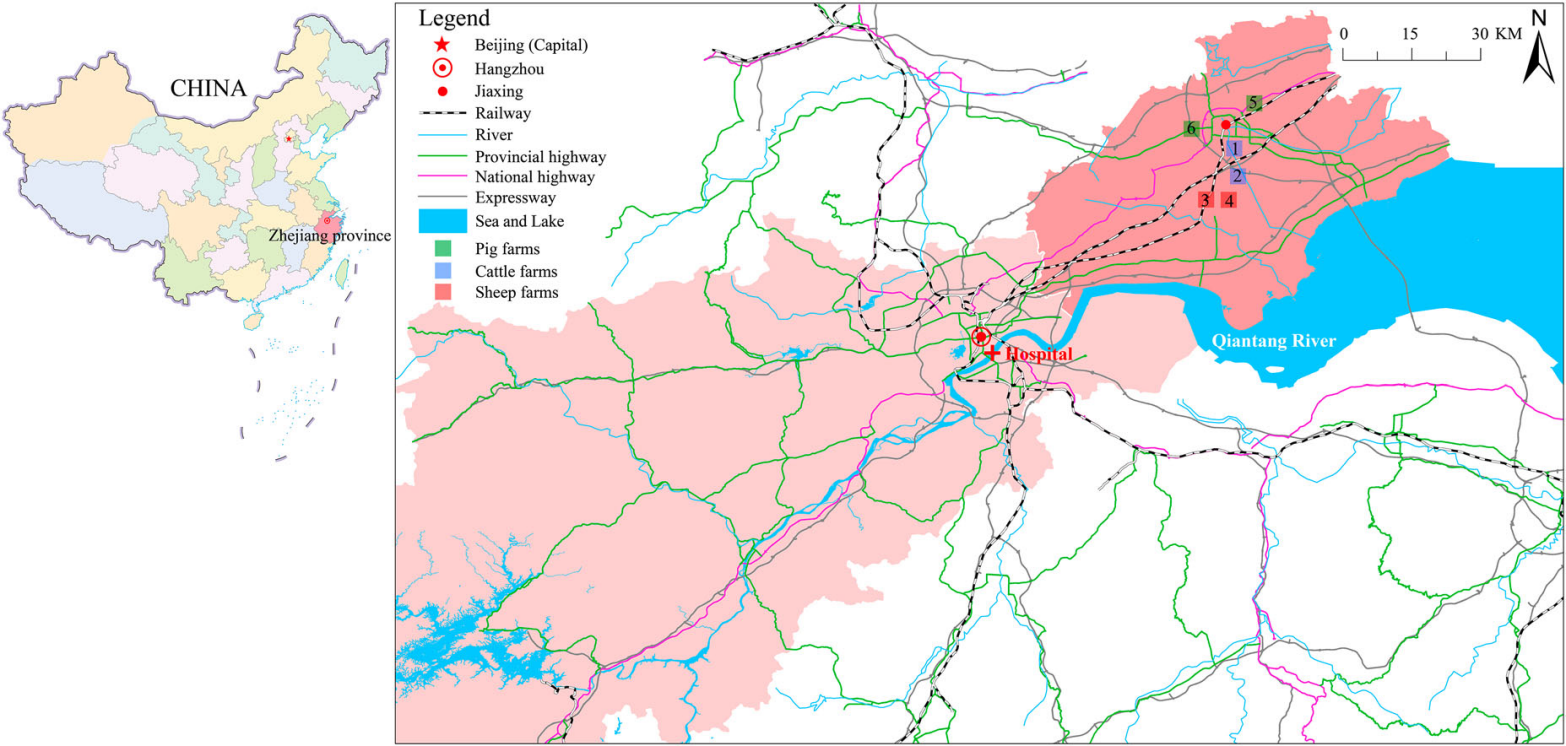
**Geographical locations of the farms and the hospital.** The map shows the local area of Zhejiang province, which is located in the east of China. The hospital is located in Hangzhou city, and farms are located in Jiaxing city. The two cities are 80 km apart and have convenient transportation, such as highways and railways. Hangzhou is the provincial capital and receives referral patients from Jiaxing. In this study, samples were collected from six farms. Blue squares represent two cattle farms (Farm 1 and Farm 2), red squares represent two sheep farms (Farm 3 and Farm 4), and green squares represent two pig farms (Farm 5 and Farm 6). Samples were collected from each farm, including animal stools, soil, vegetables, wastewater, fodder, farmer stools, and domestic environmental samples.

This prospective study was conducted in the adult ICU of the First Affiliated Hospital, Zhejiang University School of Medicine, Hangzhou, China. Patients newly admitted to the ICU from July 2018 to December 2019 were enrolled. We collected stool samples every 3 days. Sponge swab samples from the same adult ICU environment were collected during the same period every 2 weeks. The regular sampling sites included monitor panels, bed rails, floor, toilets, bedpans, hands of medical staff, handwashing sinks, keyboards, gowns, mops and beside cabinet. Diarrhea was defined as three or more loose stools within 24 h. Patients with diarrhea, whose stool samples were positive in both *C. difficile* culture and toxin gene tests, and with no evidence of other causes, were diagnosed as CDI [18]. Those with stool cultures positive for *C. difficile* and without diarrhea were defined as asymptomatic carriers. Six different animal farms that were 60–80 km away from the hospital were sampled in May 2019 (Figure 1). Stools from both animals and farmers, and swab samples from soil, wastewater, vegetables, fodder, and domestic environments, were collected. This prospective study was approved by the ethics committee of the First Affiliated Hospital, Zhejiang University School of Medicine, Hangzhou, China (No: 2016-458-1).

### Strain isolation and culture.

Due to the spore-forming properties, we treated stool samples with alcohol-shock. Stool samples were mixed with 95% ethanol and cultured on cycloserine–cefoxitin–fructose agar (CCFA, Oxoid, Basingstoke, United Kingdom) supplemented with 7% sheep blood for 48 h at 37°C with anaerobic incubation (80% N_2_, 10% H_2_, 10% CO_2_). Perianal swabs, sponge swabs, soil, wastewater, and fodder were transferred to brain heart infusion broth (BHIB) supplemented with taurocholic acid, l-cysteine, yeast extract, and cefoxitin (Oxoid, Ltd., Basingstoke, England) for enrichment. Leafy vegetables were smashed with mortar and residue was transferred to BHIB for enrichment. After anaerobic incubation for 7 days, the suspension was centrifuged at 4,000 g for 10 min, and the sediment was treated in the same manner as that for stool samples. The isolates were confirmed as *C. difficile* as previously described [19]. The presence of *tcdA*, *tcdB*, and binary toxin genes (*cdtA*, *cdtB*) was detected by PCR as previously described [20]. Toxigenic strains of *C. difficile* were defined as positive for at least one toxin gene including *tcdA*, *tcdB*, or binary toxin genes, while non-toxigenic strains were absence of all toxin genes [21].

### PCR ribotyping

PCR ribotyping was performed using capillary gel electrophoresis as previously described [22]. The 16S rRNA gene primers were labeled with carboxyfluorescein. PCR products were analyzed in an ABI 3100 genetic analyzer (Applied Biosystems, Foster City, CA, USA) with 36-cm capillary loaded with a POP4 gel (Applied Biosystems). Peak height was determined using GeneMapper ID-X 1.3 (Applied Biosystems). Gel patterns were submitted to the WEBRIBO database (https://webribo.ages.at/) for RT assignment.

### Antimicrobial susceptibility testing

Antimicrobial susceptibility testing was performed using the agar dilution method according to guidelines of the Clinical and Laboratory Standards Institute (CLSI). *C. difficile* ATCC700057 was used as the quality control strain. The following 14 antimicrobial agents were tested: metronidazole, vancomycin, clindamycin, erythromycin, amoxicillin–clavulanic acid, piperacillin–tazobactam, ceftriaxone, meropenem, moxifloxacin, levofloxacin, tetracycline, rifampicin, linezolid, and chloramphenicol. Resistance breakpoints determined by the European Committee on Antimicrobial Susceptibility Testing (EUCAST) v 10.0 were used for vancomycin (>2 µg mL^−1^), linezolid (>4 µg mL^−1^), and rifampicin (>32 µg mL^−1^) (http://www.eucast.org/clinical_breakpoints/), as no CLSI recommendations exist for these antibiotics. The breakpoint for levofloxacin was determined according to a previous study [22].

### WGS and data analysis

Genomic DNA was extracted using the FastDNA® Spin Kit for Soil (MP Biomedicals, Illkirch, France). Strains were subjected to WGS, performed on the Illumina HiSeq 2500 (Illumina, San Diego, CA, USA). The quality control of raw sequenced reads was performed using FastQC v.0.11.5 (https://www.bioinformatics.babraham.ac.uk/projects/fastqc/) and adapter regions were trimmed using Trimmomatic v.0.40 [23]. Trimmed reads were assembled de novo using SPAdes v.3.6 [24]. Multi-locus sequence typing (MLST) was performed using the mlst script (https://github.com/tseemann/mlst), and the new allele of *recA* was submitted to the pubMLST database for type assignment [25]. Genomes were submitted to the pubMLST database for MLST clade classification and allele typing of a set of genes, including S-layer cassette variants. S-layer cassette, a genetically variable 10-kb cassette, consisted with adhesion-associated genes, including *slpA*, *secA2* (encoding a secretory protein), *cwp66* (encoding an adhesin), and so on. It was characterized by diversity of those genes [26]. Genome annotations were performed on the RAST server (rast.nmpdr.org) and with Prokka [27].

### SNP calling and recombination detection

Variant calls for SNP analysis were performed using Snippy (https://github.com/tseemann/snippy) with default parameters. The chromosome of CD630 (AM180355.1) was set as the reference. The alignment file was filtered from variants with elevated densities of base substitutions as putative repetitive regions, mobile genetic elements (MGEs) and recombination events by Gubbins v.2.4.1 [28]. and used to calculate the pairwise cgSNP. The cgMLST analysis were performed using chewBBACA [29]. Clonal group (CG) was defined as strains differing by ≤ 2 cgSNPs while singleton isolate defined as the strain having not formed a CG [7]. The threshold defining CG in cgMLST was ≤ 6 SNPs [26]. The maximum likelihood trees based on core genome were constructed using MEGA11 with 1000 bootstrap replicates and visualized using the Interactive Tree of Life (iTOL) web server [30, 31]. The minimum spanning tree was constructed in PHYLOViZ 2.0 based on pairwise comparison of cgSNP and cgMLST [32].

### Identification of antimicrobial resistance genes, virulence genes, plasmids, and transposons

Antimicrobial resistance genes were identified using the BacWGSTdb server [33], and virulence genes were predicted based on the Virulence Factors of Pathogenic Bacteria Database web server (http://www.mgc.ac.cn/VFs/). Transposons were identified using BLAST, with identity and coverage requirements set to >80%. A custom sequence library was prepared as previously described [34]. Replicons of plasmids were identified using PlasmidFinder [35]. The method of *tcdB* subtyping based on neighbor-joining cluster analysis with the minimal cutoff of 5.03% dissimilarity at amino-acid level [36].

### Statistical analysis

Statistics analysis was performed using SPSS version 23.0 (SPSS, Chicago, IL, USA) and *P* value ≤ 0.05 was considered as statistical significance. Continuous variables were compared using ANOVA test or Kruskal Wallis test appropriately. Categorical data were compared using chi-square test.

## Results

### Sample collection and isolation of C. difficile

#### Sample collection and C. difficile isolation in the hospital

From June 2018 to December 2019, a total of 291 patients admitted to the adult ICU were enrolled, and 711 stool samples were collected (range, 1–24 per case). *C. difficile* was isolated from 110 stools from 57 patients, with two or more samples being positive for *C. difficile* in 21 patients. According to CDI diagnostic criteria, 55 (18.90%, 55 of 291) patients were asymptomatic carriers, of whom 32 (58.18%, 32 of 55) were colonized with *C. difficile* at admission and 23 (41.82%, 23 of 55) were colonized during hospitalization. Furthermore, two patients were diagnosed with CDI. A total of 608 environmental swab samples were collected, and 12 (1.97%) *C. difficile* strains were isolated from bedpans (4), toilets (3), mops (2), floors (2), and gowns (1). In addition, another eight *C. difficile* strains cultured from CDI patients who were hospitalized in the same ward from 2016 to 2017 were included in the study.

#### Sample collection and strain isolation at farms

During the study period, 965 samples were collected from six farms in Jiaxing city, adjacent to Hangzhou city, Zhejiang province (Figure 1). In 769 animal stool samples, 14 *C. difficile* strains were detected, eight from sheep stools of Farm 3 (3.25%, 8 of 246), one from pig stools of Farm 6 (0.55%, 1 of 182), and five from piglet perianal swab samples of Farms 5 and 6 (4.13%, 5 of 121), while *C. difficile* was not detected in cow stools. Three of 12 farmers, who worked on cattle Farm 2 (2) and pig Farm 5 (1), were positive for *C. difficile*. Of 80 soil samples, four (5%) from cattle Farm 2 (1), pig Farm 5 (1) and pig Farm 6 (2) were positive for *C. difficile*. *C. difficile* was not detected in vegetables, wastewater, fodder, or domestic environmental swab samples (Table S1).

#### MLST and RT analysis

**Figure 2.**
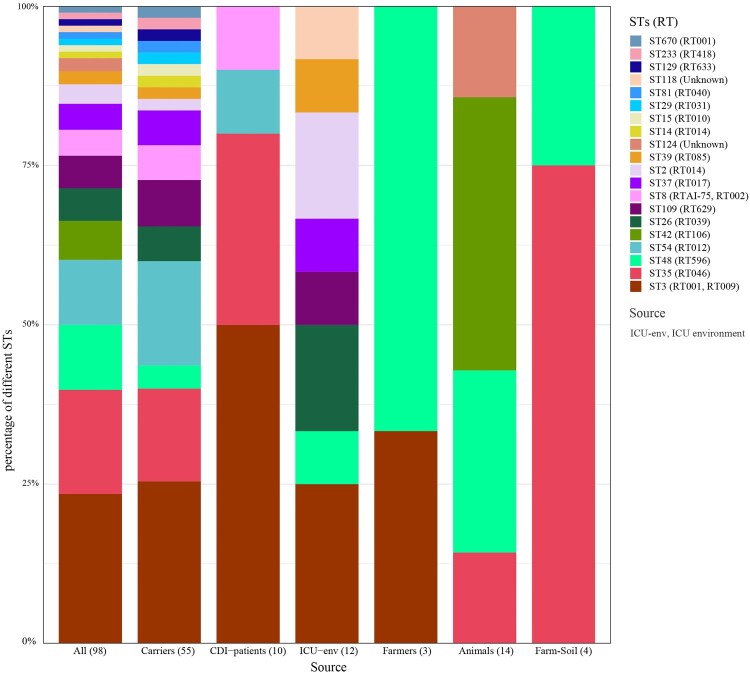
**Column chart of ST distribution from different sources.** The numbers in parenthesis represent the number of strains isolated from the respective sources.

Taken together, 98 *C. difficile* strains were assigned to 20 STs, among which ST3 was predominant (23.47%, 23/98), followed by ST35 (16.33%, 16/98) and ST48 (10.20%, 10/98). ST670 is a novel type that is a single-locus variant of ST3, differing by only one nucleotide (*recA*, T477C). STs of *C. difficile* from asymptomatic carriers and the ICU environment were more diverse. Fifty-five strains from asymptomatic carriers were assigned to 17 STs, with ST3 (25.45%, 14/55) being predominant, followed by ST54 (16.36%, 9/55) and ST35 (14.55%, 8/55). Eight STs were identified in 12 strains isolated from the ICU environment, with ST3 (25.00%, 3/12) still being dominant, among which seven were the same as those identified in carrier strains. Ten CDI strains were assigned to ST3 (5), ST35 (3), ST54 (1), and ST8 (1). Five STs were identified in 21 farm strains, including ST3 (1), ST35 (5), ST48 (7), ST42 (6), and ST124 (2). Among the above STs, ST3, ST35, and ST48 were distributed widely in four of six, four of six, and five of six sources, respectively (Figure 2).

Similarly, 98 *C. difficile* strains were assigned to 20 RTs, among which RT001 was predominant (19.39%, 19/98), followed by RT046 (16.33%, 16/98) and RT596 (10.20%, 10/98) (Table S2). The ST and RT assignments had a general agreement of 67.35% (66/98), and exclusive correlations were found in 15 STs/RTs (e.g. ST35/RT046, ST48/RT598). ST3 strains were assigned to RT001 (78.26%, 18/23) and RT009 (21.74%, 5/23), and ST8 strains were assigned to RTAI-75 (3/4) and RT002 (1/4) (Figure 2). Taken together, ST3/RT001 (18.38%, 18.98), ST35/RT046 (16.33%, 16/98) and ST48/RT596 (10.20%, 10/98) were predominant types and distributed widely in multiple sources.

#### Phylogenetic and core-genome analysis

**Figure 3.**
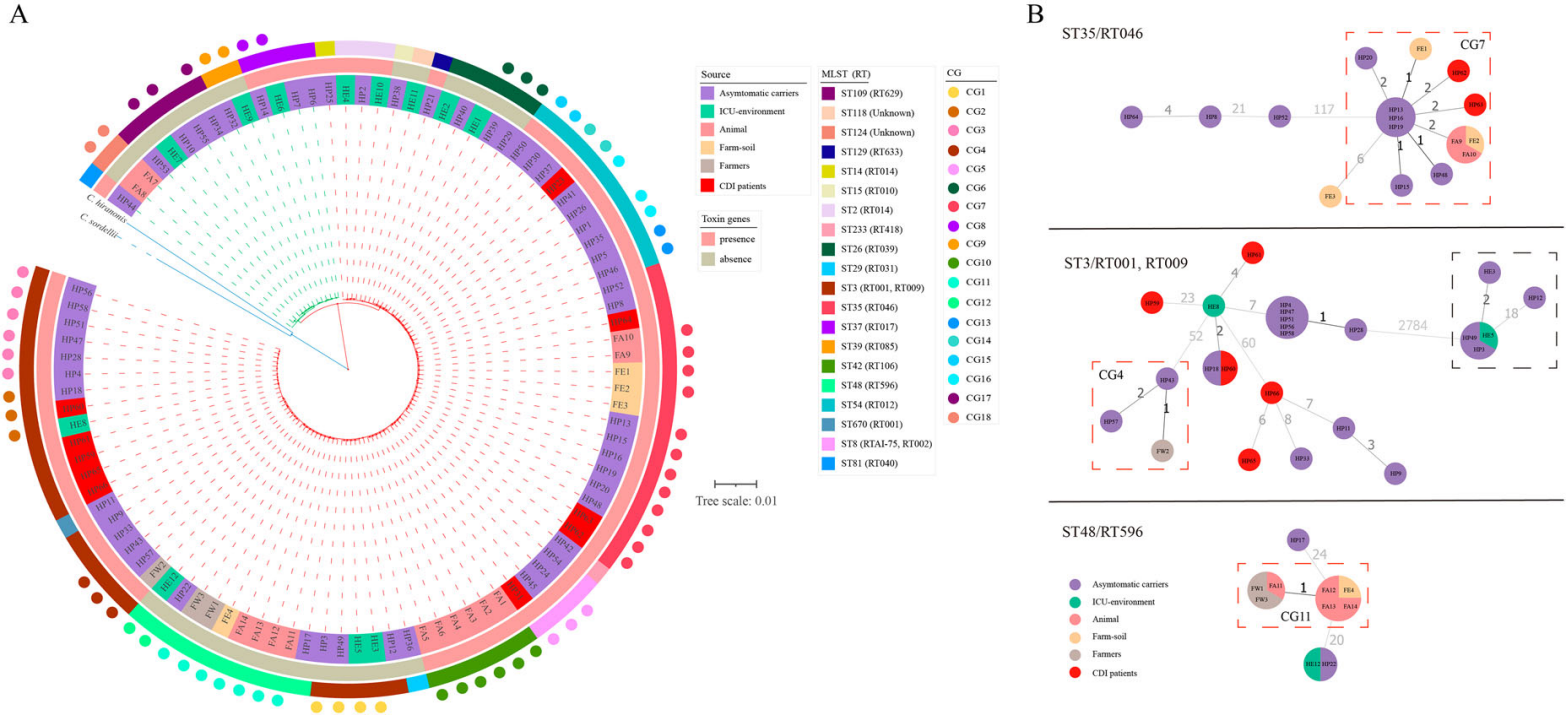
**Phylogenetic analysis and CG distribution.** A. Maximum likelihood phylogenetic tree of 98 *C. difficile* strains based on the core genome. The differently coloured branches represent two MLST clade strains: the red branches belong to MLST clade 1, and the green branches belong to MLST clade 4. The blue branches were *Clostridium hiranonis* (*C. hiranonis*) and *Clostridium sordellii* (*C. sordellii*), which were used as outgroups to root the tree. Coloured rings from the inside out represent the sources of the strains, presence or absence of toxin genes, and STs. Coloured dots represent the distribution of CGs. B. Minimum spanning trees of strains in ST35/RT046, ST3/RT001, RT009, and ST48/RT596. The colours of the circles represent the sources, and sizes of the circles are related to the number of strains in the CG: the more strains in the CG, the larger the size. Values between the circles are the number of cgSNPs, where smaller numbers are indicated by values with darker colour. Dashed red square boxes are CGs comprising strains from multiple sources. Strains in dashed black square boxes are non-toxigenic ST3/RT009 strains, while the rest of the ST3/RT001 strains are toxigenic. HP, HE, FA, FE, and FW are strain names, representing strains from hospitalized patients in the ICU including patients with CDI and asymptomatic carriers, the ICU environment, animals, soil, and farmers, respectively.

A phylogenetic tree was constructed based on the core-genome. Ninety-eight *C. difficile* genomes were divided into two MLST clades, MLST clade 1 (85.71%, 84 of 98) and MLST clade 4 (14.29%, 14 of 98). The phylogenetic tree revealed multiple evolutionary clusters that were broadly congruent with ST lineages and toxin gene profiles, but independent of sample sources. Of note, ST3 strains separated into two clusters, a toxigenic cluster (ST3/RT001) and a non-toxigenic cluster (ST3/RT009) (Figure 3).

**Figure 4.**
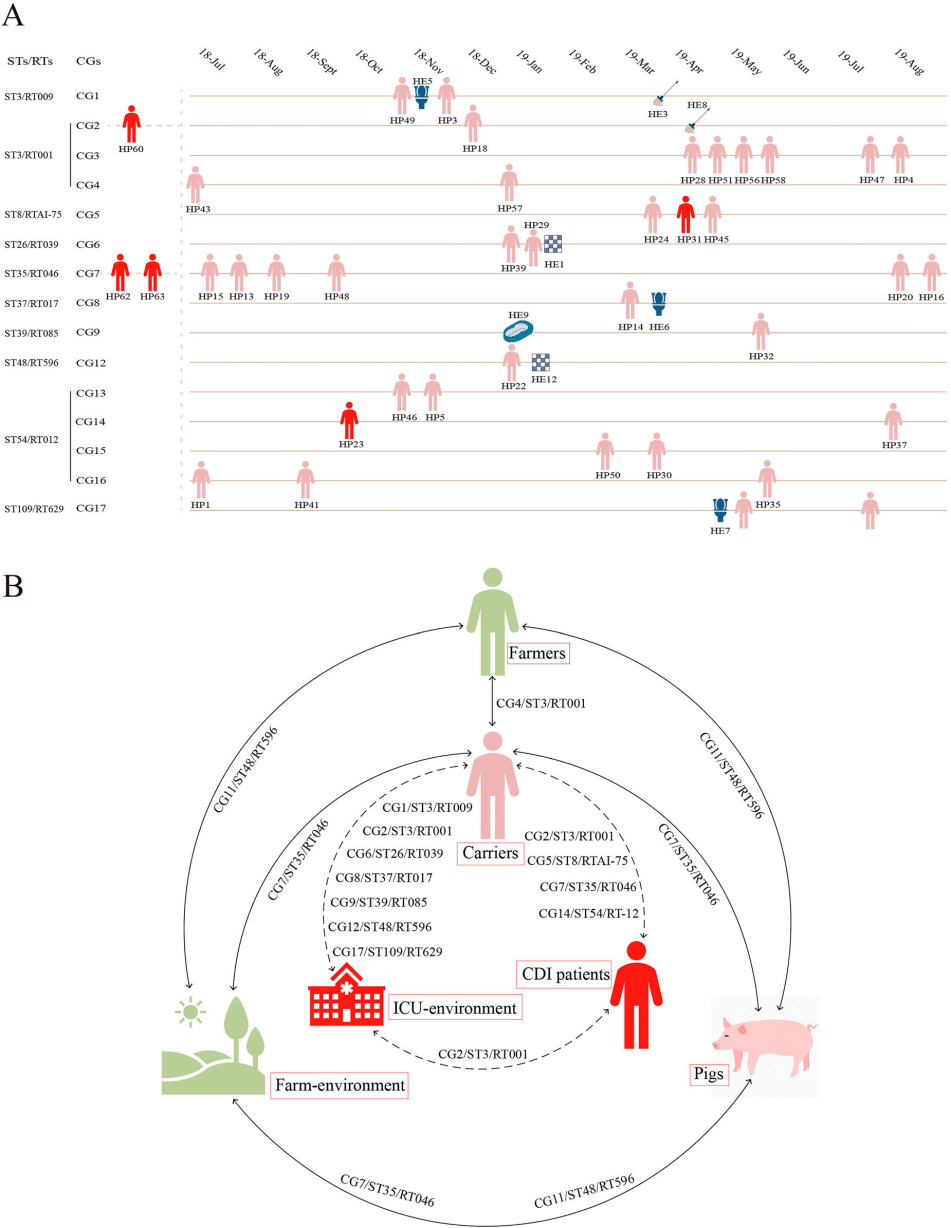
**Transmission links.** A. *C. difficile* transmission pattern in the hospital. The date on the top represents the time the strain was isolated, and each strain is placed in the corresponding month, with horizontal distances between strains representing the time difference in isolation. Strains in the same yellow line belong to the same CG and ST/RT. The pink human icons represent asymptomatic carriers, and the red icons represent patients with CDI. HP and HE represent strains sourced from hospitalized patients and the ICU environment, respectively. Strains HE5, HE6, and HE7 were isolated from the toilet, HE3 and HE8 were isolated from the mop, HE2 and HE12 were isolated from the floor, and HE9 was isolated from a gown. The three strains from patients with CDI to the left of the yellow dashed line were isolated from 2016 to 2017. B. Transmission pattern of *C. difficile* strains from different sources. The dashed lines indicate transmission links in the hospital, and the solid lines indicate transmission links between the farms and the hospital. The CGs/STs/RTs near the lines are shared by the sources at both ends of the lines. ST, sequence type; RT, ribotype; CG, clonal group.

Core-genome single-nucleotide polymorphism (cgSNP) analysis identified 14,515 SNPs. The cgSNP range varied across different STs (Fig. S1), while ST3 (0–2,802) had the widest range, followed by ST35 (0–128). A minimal spanning tree was constructed for multi-source strains with ST35/RT046, ST3/RT001, RT009, and ST48/RT596 (≥ 3 sources), and we found that the strains from different sources were closely related, as numbers of cgSNPs were less than two or even zero (Figure 3(b)). Clonal group (CG) was defined as strains differing by ≤ 2 cgSNPs. The definition of singleton isolate was that the strain differed by > 2 cgSNPs with any other strains and have not formed a CG. Thirty singleton isolates and 18 CGs were identified across 98 genomes. Of 18, 13 CGs merely consisted of hospital strains, and three CGs merely consisted of farm strains, while two CGs consisted of both farm and hospital strains (Figure 3). Of 13 hospital CGs, six CGs comprised asymptomatic carrier and ICU environment strains, four CGs comprised asymptomatic carrier strains, two CGs comprised CDI patient and carrier strains, and one CG comprised CDI patient, carrier, and environment strains (Figure 4(a)). Of note, one farm CG consisted of farmer, pig, and soil strains.

Seven singleton isolates and four CGs (CG1–CG4) were identified in 23 ST3 strains. CG1–CG3 were hospital CGs; CG1 consisted of asymptomatic carrier and ICU environment strains; CG2 consisted of CDI patient, asymptomatic carrier and ICU environment strains and CG3 consisted of six carrier strains; while CG4, a farm and hospital CG, consisted of one farmer and two carrier strains. Four singleton isolates and one CG (CG7) were identified in 16 ST35/RT046 strains. CG7, the largest CG in this study, consisted of six carrier, two CDI patient, two soil (Farm 2 and Farm 5), and two pig strains (Farm 5). One singleton isolate and two CGs (CG11 and CG12) were identified in ten ST48/RT596 strains. CG11, a farm CG, consisted of two farmer (Farm 2 and Farm 5), one soil (Farm 6), and four pig strains (Farm 6). CG12, a hospital CG, consisted of one carrier strain and one ICU environment strain. Pairwise comparison of cgSNPs between CG11 and CG12 was 20–21 (Figures 3(b) and 4(b)).

We also analyzed the genetic relationships of multi-source strains based on core genome multi-locus sequence type (cgMLST). The CG composition and distribution of cgMLST were accordance with cgSNP (Fig. S2).

#### Comparison of virulence genes in multi-source C. difficile strains

Among 98 strains, there were 66 toxigenic strains (67.35%), including 61 (62.24%) strains positive for both *tcdA* and *tcdB* (A^+^B^+^), and five (5.10%) *tcdA*-negative and *tcdB*-positive (A^−^B^+^) strains, while no strains were positive for binary toxin genes (CDT^−^). All A^−^B^+^CDT^−^ strains were ST37/RT017 and ST81/RT040 and belonged to MLST clade 4, while all A^+^B^+^CDT^−^ strains belonged to MLST clade 1 (Figure 3). There was no significant difference in the proportion of toxigenic strains between farm and hospital strains (57.14% versus 70.13%, *X*^2^ = 1.265, *P* = 0.261). The genetic environment of the pathogenic locus (PaLoc) was very conserved across all toxigenic strains, comprising *tcdA*, *tcdB*, *tcdC*, *tcdE*, and *tcdR* genes, except for five A^−^B^+^CDT^−^ strains lacking *tcdA* (Fig. S3). According to the type method of *tcdB*, two types were identified, including tcdB1 and tcdB3. All animal, farmer, soil, and CDI patient strains, and most asymptomatic carrier (36 of 40) and ICU environment strains (3 of 4), were type tcdB1; only five ST37/RT017 and ST81/RT040 strains from asymptomatic carriers and the ICU environment were type tcdB3. Toxigenic regulation genes, including *tcdC*, *tcdR*, and *tcdE*, were highly conserved across multi-source strains in the same ST (ST3, ST35, ST48) with 100% identity. The type of S-layer cassette, an adhesion-associated gene cluster, was highly correlated with MLST and toxin gene profiles, but independent of sources. ST35/RT046 strains were type 3, ST48/RT596 strains were type 7, toxigenic ST3/RT001 strains were type 4, and non-toxigenic ST3/RT009 strains were type 8 (Table S2).

**Figure 5.**
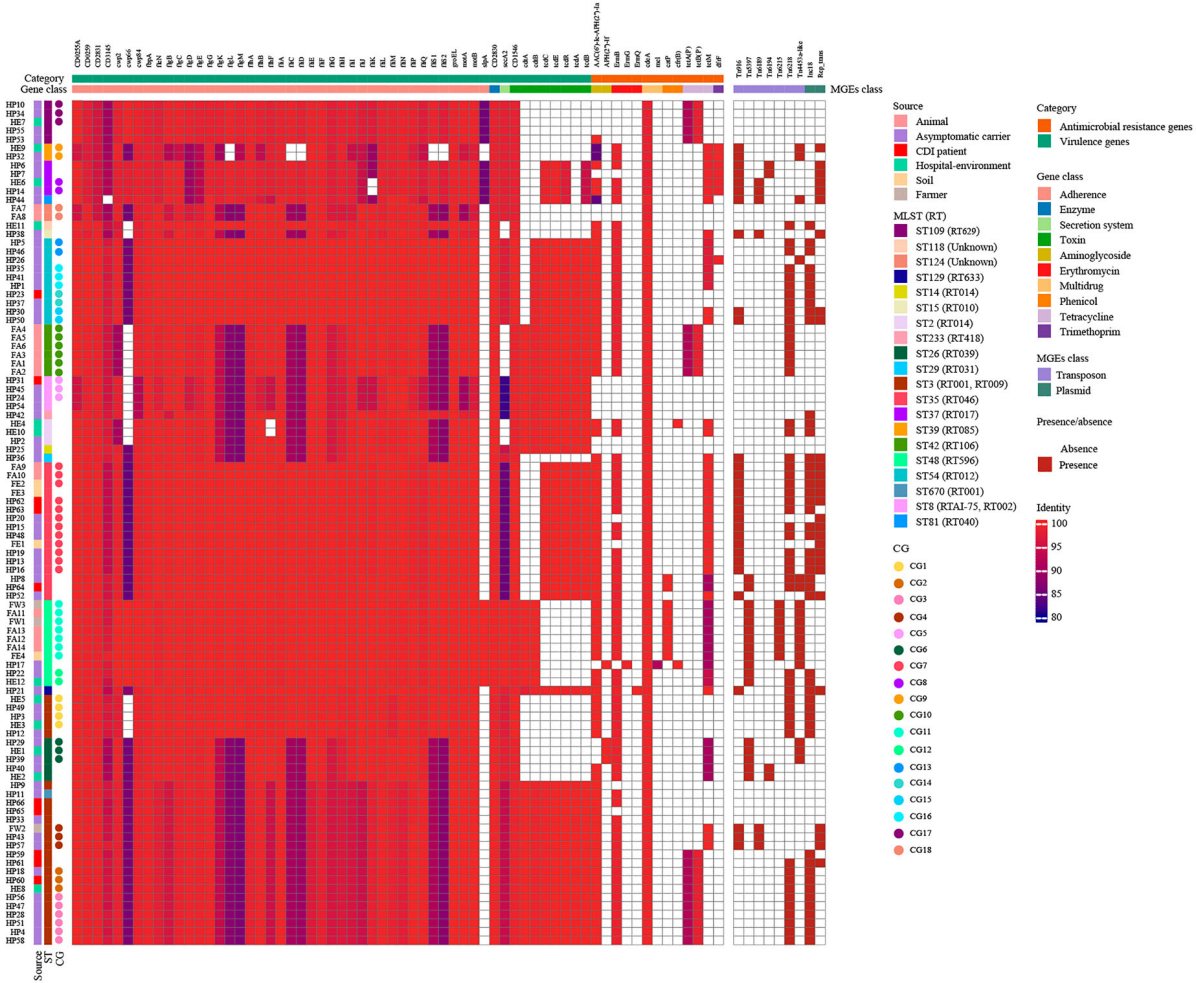
**Virulence genes, resistance genes, transposons, and plasmid type comparison.** The named category in the top row represents virulence genes and resistance genes, while the gene class in the second row represents the functional classification of the corresponding virulence genes and resistance genes. Columns from left to right represent the strain names, sources, STs, and CGs. We set the identity above 80% for the presence of genes and below 80% for the absence of genes. Colours of squares below gene class indicate identity values of genes, while colours of those below MGEs class indicate presence or absence, with red representing presence and white representing absence.

The carriage of other virulence genes in *C. difficile* was investigated further based on the Virulence Factor Database (Figure 5). A total of 4,612 virulence genes were identified in 98 strains, with a median of 48 virulence genes per strain (range, 38–50). Of these, flagellar genes were the most numerous virulence genes with a total of 2,828 genes annotated and a median of 29 flagellar genes per strain (range, 24–29). Compared with animal, soil, farmer, asymptomatic carrier, and CDI patient strains, ICU environment strains carried fewer virulence genes (ANOVA, *P* = 0.008) and flagellar genes (Kruskal–Wallis, *P *= 0.002). The number of virulence genes carried by strains in the same CG was consistent [CG7/ST35/RT046 (48), CG11/ST48/RT596 (46), CG4/ST3/RT001 (50)]. Variations in the nucleotide sequence of virulence genes were associated with MLST and were highly conserved in strains of the same ST (ST3, ST35, ST48) (identity range, 96.46–100%), especially in strains of the same CG (identity, 100%).

#### Comparison of antimicrobial resistance phenotypes and genotypes of multi-source C. difficile strains

The distribution of minimum inhibitory concentrations of 14 antimicrobial agents against 98 strains is shown in Table S3. The highest resistance rate was observed for erythromycin and clindamycin (80.61%, 79 of 98). Compared with farm strains, hospital strains had a higher resistance rate to moxifloxacin (35.1% versus 4.8%, *X*^2^ = 7.42, *P* = 0.006) and a lower resistance rate to tetracycline (24.7% versus 90.5%, *X*^2^ = 30.09, *P* < 0.0001). In this study, strains carrying *ermB*, *tetM*, *AAC(6’)-Ie-APH(2'’)-Ia*, or *cdeA* were distributed in all sources. Resistance genes *tetA(P)* and *tetB(P)* were present in animal, ICU environment, carrier, and CDI patient strains (Figure 5). Multi-source strains in the same CG (CG7/ST35/RT046, CG11/ST48/RT596, CG4/ST3/RT001, etc.) harbored a concordant resistance phenotype and resistance gene profile with 100% identity, except for two strains from soil and asymptomatic carriers in CG7 lacking *ermB*.

To provide a genomic context for antimicrobial resistance genes, draft genomes were screened for the presence of transposons (Figure 5). Transposons carrying *ermB* include Tn*6189* (8.97%, 7 of 78), Tn*6194* (5.13%, 4 of 78), Tn*6215* (8.97%, 7 of 78), and Tn*6218* (64.10%, 50 of 78). Tn*6218* was widely distributed in strains from different sources. Tn*6215* was identified only in CG11/ST48/RT596, while it was absent in ST48/RT596 strains from asymptomatic carriers and the ICU environment. Tn*4453a* carrying *catP* had a similar distribution to that of Tn*6215*. The most common transposons carrying *tetM* were Tn*916* (53.70%, 29 of 54) and Tn*5397* (31.48%, 17 of 54), which were widely distributed among strains from various sources. Multi-source strains in CG7/ST35/RT046 carried both Tn*916* and Tn*6218*, except for two strains lacking *ermB*. Both farmer and asymptomatic carrier strains in CG4/ST3/RT001 carried Tn*916* and Tn*6189*, while CDI patient, asymptomatic carrier, and ICU environment strains in CG1/ST3/RT009 and CG2/ST3/RT001 carried Tn*6218*.

Two plasmid incompatibility groups were identified across 98 strains, including Inc18 (47.96%, 47 of 98) and Rep_trans (28.57%, 28 of 98). Multi-source strains in CG7/ST35/RT046 carried both Rep_trans and Inc18 plasmids, except that only one soil strain carried the Rep_trans plasmid. Of the ST3 strains, all CG4 strains carried the Rep_trans plasmid, while both CG1 and CG2 strains carried Inc18. Of the ST48 strains, none of the seven strains in CG11 carried plasmids, while two strains from a carrier and the ICU environment in CG12 carried the Inc18 plasmid.

## Discussion

This study prospectively investigated *C. difficile* strains isolated from multiple sources in China. To a certain extent, this study elucidates the molecular epidemiological characteristics of *C. difficile* from different sources, and, for the first time, genetic relationships are analyzed between *C. difficile* strains from different sources with both molecular epidemiological and genomic perspectives. This study also demonstrates the phenomenon that *C. difficile* transmission occurs between animals, the environment, farmers, symptomatic carriers and patients.

In the past two decades, the incidence of CA-CDI increased significantly [5]. Risk factors for CA-CDI were not the same as those previously identified for HA-CDI, suggesting that the source of CDI is in the broader community rather than in healthcare facilities [37]. In 2013, Eyre et al. [7] found that 45% of isolates from CDI cases were genetically distinct from those from previous CDI cases (cgSNPs > 10), and only 35% of isolates were related (cgSNP ≤ 2), suggesting that diverse sources, in addition to symptomatic patients, play a major role in *C. difficile* transmission. In addition, a study conducted by Gonzalez-Orta et al. [38] showed that more than a quarter of patients diagnosed with HA-CDI at Cleveland Hospital in the United States were infected with strains that colonized on admission. Subsequently, studies found that asymptomatic hospitalized carriers are a potential source for transmission of CDI strains among long-term care facility residents [6, 8]. These studies suggest that *C. difficile* in the community is an important reservoir of CDI strains.

CA-CDI and *C. difficile* colonization of asymptomatic carriers may be associated with exposure to *C. difficile* from different sources in the community, such as domestic animals, which could be a possible source of infection for CA-CDI. Previous studies showed that both toxigenic and non-toxigenic *C. difficile* can be isolated from intestines of animals, such as piglets, cattle, and farmers, and be disseminated through direct contact, the food chain, and the environment [11, 39]. In this study, 25% of farmers were colonized with *C. difficile*, which is consistent with the 25% intestinal colonization rate of Dutch pig farmers reported by Keessen et al. [40]. Although it is not clear whether the incidence of CDI in farmers differs from that in other populations, and further research is needed, there is no doubt that these asymptomatic carriers can act as a source of CDI strains. In this study, MLST and RT analysis of *C. difficile* strains from different sources revealed that strains belonged to ST3/RT001, ST35/RT046 and ST48/RT596 were distributed widely in multiple sources, including farmers, asymptomatic carriers, symptomatic CDI patients, and animals. Among these strains, ST3 and ST35 were the main prevalent STs in China [19]. Multicenter survey showed ST3/RT001 was an epidemic strain in some European countries and ST35/RT046 was prevalent CDI strain in Sweden and Asia-Pacific region [15, 41, 42]. Studies focusing on multiple sources found that ST3/RT001 was prevalent in broiler manure and chicken, and ST35/RT046 was highly prevalent in piglets [11, 15, 43]. These studies suggest that *C. difficile* transmission between humans and animals may occur via the food chain.

To further investigate genetic relationships of *C. difficile* strains from different sources, we performed phylogenetic and core-genome analyses. A total of 18 CGs were identified, among which 13 CGs merely comprised hospital strains, and three CGs comprised farm strains, while two CGs (CG7/ST35/RT046 and CG4/ST3/RT001) comprised both farm and hospital strains (Figures 3 and 4). CG7/ST35/RT046 comprised 12 multi-source *C. difficile* strains, including six carrier, two CDI patient, two soil (Farm 2 and Farm 5), and two pig strains (Farm 5), all of which were type tcdB1 for *tcdB* and type 3 for S-layer cassette. All strains in CG7 possessed the same virulence and resistance gene profile with similar resistance phenotypes, harbored identical transposons (Tn*916* and Tn*6218*), and carried plasmids typed as Rep_trans and Inc18. This suggests that no significant genomic differences exist between *C. difficile* strains from CDI patients, asymptomatic carriers, pigs, and soil. The same phenomenon was observed for CG4/ST3/RT001, although this CG only comprised one carrier and one farmer strain. CG11/ST48/RT596 was a farm CG comprising multi-source strains, including farmer (Farm 2 and Farm 5), soil (Farm 6), and pig strains (Farm 6). Although the strains in this CG were non-toxigenic, the virulence gene profile, the resistance gene profile, the resistance phenotype, and even the structure of the transposon carrying *ermB* (Tn*6215*) were almost identical, and none of them carried the plasmid. This suggests that no significant genomic differences exist between *C. difficile* strains from farmers, soil, and pigs. Frentrup et al. found that RT001 strains isolated from broiler manure were genetic associated with human isolates [44]. Werner et al. confirmed that RT046 strains isolated from piglets and environment were closely related with human isolates [15]. The comprehensive comparison of global multi-source strains in this study demonstrated that the close molecular epidemiology of multi-source strains was also observed in global context (Fig S4).

The hospital selected for this study is the largest general teaching hospital in the region, while the farms are <100 km away from the hospital. In addition, there is convenient transportation, with movement of a large population between the two cities, and populations overlap in several aspects such as hospital visits, the food chain, and civilian mobility. This suggests that *C. difficile* does not exist exclusively in single source, and there is indeed a close molecular epidemiological correlation between various sources, which does not even exclude interspecies and cross-regional transmission of *C. difficile* between communities and hospitals. Based on epidemiological data, we speculate that a plausible transmission pattern of *C. difficile* exists between animals, the environment, farmers, asymptomatic carriers and CDI patients. First, fecal–oral transmission may occur through contaminated meat and vegetables products. Previous studies detected *C. difficile* in retail meat and root vegetables [16, 43, 45], although no meat samples were collected in this study to provide direct evidence. Second, as farmers are highly mobile and move freely between communities and hospitals, it is reasonable that farmers play an important role as vectors in *C. difficile* transmission via contact with other living organisms, common environmental exposure, and migration [46, 47]. Ultimately, this leads to interspecies and cross-regional transmission of multi-source *C. difficile* strains between animals, the environment, farmers, asymptomatic carriers and CDI patients.

Asymptomatic carriers deserve more attention in this study, as 83.33% (15/18) of CGs comprised asymptomatic carrier strains, suggesting that asymptomatic carriers play a bridging role in the transmission of *C. difficile*. Meanwhile, among the 13 hospital CGs, six CGs comprised carrier and ICU environment strains, four CGs comprised asymptomatic carrier strains, two CGs comprised carrier and CDI patient strains, and one CG comprised carrier, CDI patient, and ICU environment strains (Figure 4). Strains in the same CG possessed consistent virulence and resistance gene profiles, with similar resistance phenotypes, and harbored identical transposons and plasmid types (Figure 5, Table S3), which suggests that CDI patient, carrier, and ICU environment strains are genetically related. Two recent studies showed that asymptomatic carriers can cause epidemics and transmission of CDI in hospitals [48, 49]. In separate studies conducted by Halstead et al. [48] and Sheth et al. [49], the asymptomatic carriage rate of *C. difficile* was around 10–15%, with over 80% of patients colonized with toxigenic strains. More importantly, genetic testing techniques revealed that *C. difficile* can be transmitted from asymptomatic carriers to other patients. This study also demonstrates that, in addition to CDI patients, asymptomatic hospitalized carriers play an important role as transmission vectors in *C. difficile* dissemination in hospitals.

This is a prospective study, and the farms selected in this study were limited to one surrounding city, resulting in a potentially uneven collection of specimens. However, considering that the city is the main livestock production base, this limitation is compensated to some extent. Based on the entire food chain, the transmission pattern of *C. difficile* from farms to hospitals can be explored more completely by testing meat on sale in markets. Regrettably, meat products were not collected in this study. In addition, because of the small number of CDI strains included in this study, the transmission pattern of strains from symptomatic CDI patients, asymptomatic carriers, and the ICU environment cannot be fully demonstrated.

This is the first study to investigate the variability and correlation between strains from different sources at both phenotypic and genomic levels in China. It enriches epidemiological data of *C. difficile* in farms and asymptomatic carriers. This study found that interspecies and cross-regional transmission of *C. difficile* occurs between animals, the environment, farmers, asymptomatic carriers and CDI patients, and does not rule out the possibility that farm strains, including those from animals, farmers, and soil, as well as those from asymptomatic hospitalized carriers, are important reservoirs of CDI strains. As a link for *C. difficile* transmission between the community and hospitals, asymptomatic hospitalized carriers are very important for the dissemination of *C. difficile*.

## Supplementary Material

supplementary_file-revised-clean-version.docx

## Data Availability

This whole-genome shotgun project of 98 *C. difficile* isolates was submitted to GenBank under accession numbers JACFUE000000000-JACFXP000000000 and JAGDLT000000000-JAGDMA000000000 (Table S2). The version described in this paper is the first version.
