# Supplementary material for: Comparative genomic and transmission analysis of Clostridioides difficile between environmental, animal, and clinical sources in China
Source: Emerg Microbes Infect. 2021 Dec 1;10(1):2244–55. doi: 10.1080/22221751.2021.2005453 (PMC8648027; doi:10.1080/22221751.2021.2005453)
Supplement: supplementary_file-revised-clean-version.docx [file TEMI_A_2005453_SM1223.docx]

**Comparative genomic and transmission analysis of *Clostridioides difficile* between environmental, animal, and clinical sources in China: Appendices**

**Materials and Methods**

*Retrieve and analysis of global genomes of C. difficile in the database.*

We downloaded all assembled genomes (3,864) of *C. difficile* deposited in GenBank (as of 14th May 2021) and 47 raw reads data sets in the ENA database according to the literature [1]. The raw reads were assembled using SPAdes v.3.6 [2]. In total, 3,911 global genomes were used to screen for the ST3, ST35 and ST48 strains. WGS analysis of global genomes was performed as described above.

**Result**

***Comparison of multi-source C. difficile strains (ST3, ST35, ST48) in global context.***

Comparison of multi-source *C. difficile* strains (ST3, ST35, ST48) in the global context. To further understand the global dissemination of multi-source strains, we identified 129 ST3 strains, 84 ST35 strains and 25 ST48 strains in 3,911 genomes retrieved from GenBank and the European Nucleotide Archive (ENA) database. Global strains were also distributed widely in the environment (24 isolates), animals (41), and humans (173), with 39 strains being of unknown source (Table S2). A maximum likelihood phylogenetic tree was constructed for 277 genomes consisting of strains from China (50) and the globe (227). The phylogenetic tree showed that strains were congruent with ST lineages and toxin gene profiles, but independent of sample sources and countries. Furthermore, strains from China were interspersed among the global strains (Fig. S4A).

A total of 195 (70.40%) strains were toxigenic among 277 strains, including A^+^B^+^ (64.26%, 178 of 277) and A^−^B^+^ (6.14%, 17 of 277) strains, all of which were CDT^−^. There were no significant differences in the proportion of toxigenic strains of ST35 (100%) and ST48 (*X^2^*= 4.861, *P* = 0.182) between different sources. But the proportion of toxigenic strains in ST3 from the environment was lower than those that from humans and animals (16.67% versus 66.07% versus 91.67%, *X^2^* = 10.661, *P* = 0.014). The genetic environment of the PaLoc was very conserved across all A^+^B^+^CDT^−^ toxigenic strains, except for three human ST3 strains (from the USA), which were negative for *tcdR*. The *tcdB* sequences were very conserved (identity 99.93%–100%) across multi-source strains from different countries in the same ST. The sequence of toxigenic regulation genes was also conserved across multi-source strains in the same ST, especially those for *tcdR* (99.56%–100%) (Fig. S4B). The S_layer_cassette type of ST35 strains from different countries and sources was type 3, that of ST48 strains was type 7, that of toxigenic ST3 strains was type 4, and that of non-toxigenic ST3 strains was type 8 (Table S2). There was no significant difference in the number of virulence genes between different sources of global strains (Kruskal–Wallis, *P* = 0.171). The identity of virulence gene sequences in multi-source strains from the same ST ranged from 87.18% to 100% (Fig. S4B).

We further analyzed the distribution of antimicrobial resistance genes and mobile genetic elements (MGEs) in global multi-source strains. Resistance genes, such as *ermB*, *tetM* and *cfr(B)*, were distributed widely in environmental, animal, and human strains. The sequence identity of antimicrobial resistance genes in multi-source strain from the same ST ranged from 84.06% to 100%. Resistance genes carrying transposons, such as Tn*916*, Tn*6218* and Tn*4453a*, were also presented in global multi-source strains. Four plasmid incompatibility groups were identified across 277 strains, with Inc18 and Rep_trans being identified in multiple sources (Fig. S4B).

**Reference**

1. Werner A, Molling P, Fagerstrom A, et al. Whole genome sequencing of *Clostridioides difficile* PCR ribotype 046 suggests transmission between pigs and humans. PLoS One 2020. 15. e0244227. DOI:10.1371/journal.pone.0244227

2. Nurk S, Bankevich A, Antipov D, et al. Assembling single-cell genomes and mini-metagenomes from chimeric MDA products. J Comput Biol 2013. 20. 714-737. DOI:10.1089/cmb.2013.0084

**Appendices Figure legends**

**Fig. S1. Box plot diagram of SNP counts.** SNP counts were based on pairwise comparison of SNPs in the core genomes. The ordinate denotes SNP counts, and the abscissa represents STs. Because the pairwise comparisons of SNPs of six ST42 strains were zero, the SNP-count box plot diagram overlaps with the abscissa. SNP, single-nucleotide polymorphism.

**Fig. S2. Minimum spanning trees of 98 *C. difficile* strains based on cgMLST.** The colors of the circles represent the sources, and sizes of the circles are related to the number of strains in the CG: the more strains in the CG, the larger the size. Values between the circles are the number of SNPs based on cgMLST. The lines were shadowed in gray if the SNPs ≤ 6 (the threshold defined CG in cgMLST). HP, HE, FA, FE, and FW are strain names, representing strains from hospitalized patients in the ICU including patients with CDI and asymptomatic carriers, the ICU environment, animals, soil, and farmers, respectively.

**Fig. S3. Genetic environment comparison of PaLoc in ST35 and ST3 strains.** Arrows indicate the positions and directions of the genes, with gene names placed at the top. Green arrows represent virulence genes in PaLoc, and blue arrows represent genes located upstream and downstream of PaLoc. Labels from left to right are STs/RTs, CGs, and sources of strains. Red shades denote shared regions with a high degree of homology. PaLoc, pathogenic locus.

**Fig. S4. Comparison multi-sources strains (ST3, ST35, ST48) in global context.** A. Maximum likelihood phylogenetic tree of 277 *C. difficile* strains based on the core genome. The differently colored branches represent three MLST strains: the brown branches belong to ST3, the green branches belong to ST48 and the red branches belong to ST35. The blue branches were strain M120 (ST11, FN665653.1) and strain R20291 (ST1, FN545816.1), which were used as outgroups to root the tree. Colored rings from the inside out represent the sources of the strains, presence or absence of toxin genes, and countries. B. Virulence genes, resistance genes, transposons, and plasmid type comparison. The named category in the top row represents virulence genes and resistance genes, while the gene class in the second row represents the functional classification of the corresponding virulence genes and resistance genes. Columns from left to right represent the strain names, STs, sources, and countries. We set the identity above 80% for the presence of genes and below 80% for the absence of genes. Colors of squares below gene class indicate identity values of genes, while colors of those below MGEs class indicate presence or absence, with red representing presence and white representing absence.

**Table S1. Isolation of *C. difficile* from farms.** ‘-’, for the limited sample size of vegetables and fodders, it is not accurate to calculate the isolation rate.

**Table. S2. Virulence gene typing and genome assembly accession of *C. difficile* strains.** ST, sequence type; tcdB-BDR, tcdB-binding region; ‘-’, not identified or typed by the pubMLST database; the numbers indicate the gene allele type identified in the pubMLST database.

**Table. S3. MIC distribution of 98 *C. difficile* strains.** MIC, minimum inhibitory concentration; R rate, resistance rate of antimicrobial. ‘-’, statistical analysis was not performed as the resistance rates were consistent between the two groups.
